# Prevalence and factors associated with adverse birth outcomes among women with chronic hypertension in Rangpur: A multi-center cross-sectional study

**DOI:** 10.1371/journal.pone.0337526

**Published:** 2025-12-11

**Authors:** Azaz Bin Sharif, Syed Sharaf Ahmed Chowdhury, Md. Zakir Hossain, Md. Anwar Hossain, Ahmed Hossain, Hasan Mahmud Reza

**Affiliations:** 1 Global Health Institute, North South University, Dhaka, Bangladesh; 2 Department of Public Health, North South University, Dhaka, Bangladesh; 3 Hypertension and Research Center, Rangpur, Bangladesh; 4 College of Health Sciences, University of Sharjah, Sharjah, United Arab Emirates; 5 Department of Pharmaceutical Sciences, North South University, Dhaka, Bangladesh; University of North carolina at Greensboro, UNITED STATES OF AMERICA

## Abstract

**Background:**

Hypertension affects approximately 3–5% of pregnancies globally, with an increased risk of adverse birth outcomes (ABOs) among mothers with chronic hypertension, which poses a significant public health challenge. Despite global evidence, there is a lack of studies in Bangladesh specifically investigating the impact of chronic hypertension on birth outcomes. Therefore, the study aims to find the prevalence and factors associated with adverse birth outcomes among women with chronic hypertension who delivered in health facilities.

**Methods:**

An institution-based multi-center cross-sectional study was conducted in 3 different maternity and child hospitals in Rangpur City between June 2023 and September 2023 and recruited 342 hypertensive females who gave birth in an institutional setting within the last 6 months. The outcome variable was the adverse birth outcomes. For the statistical analysis, STATA version 17.0 was used. Descriptive analysis, Pearson’s chi-square test, and both the bivariable and multivariable binary logistic regression analyses were conducted to determine the association between birth outcomes and covariates.

**Result:**

The overall prevalence of adverse birth outcomes among the study participants was 36% (95% CI: 30.87, 41.30). Among the study participants, mothers belonging to a family with higher income (AOR: 3.09; 95% CI: 1.51, 6.30), participants with complicated pregnancies (AOR: 2.55; 95% CI: 1.55, 4.21), mothers who had at least one danger sign present in their last pregnancy (AOR: 2.21; 95% CI: 1.17, 4.15), exposure to secondhand smoke during their pregnancy (AOR: 3.33; 95% CI: 1.16, 9.60) were significantly associated with adverse birth outcomes among hypertensive pregnant women.

**Conclusion:**

This study observed a high prevalence of adverse birth outcomes among pregnant women with chronic hypertension who had an institutional delivery. The finding warrants immediate community-based action focused on the important factors to reduce adverse birth outcomes among hypertensive mothers. Further longitudinal studies are needed to identify any causal association between important variables and adverse birth outcomes.

## Introduction

Adverse birth outcomes (ABOs) pose significant global public health challenges. In the last 20 years, while noteworthy progress has been made in reducing child mortality [1] with a higher reduction in the under-five mortality rate, neonatal mortality has declined at a slower pace during this period. Nearly half of the global under-five mortality is contributed by neonatal deaths [2], and 75% of these are from ABOs [3]. The major ABOs include prematurity (11%), low birth weight (27%), intrapartum-related deaths (13.9 per 1000 births), and severe neonatal infections [4–6]. The global incidence of ABOs has decreased significantly in recent decades. However, disparities persist between high-income and low- to middle-income countries (LMICs) [7]. While the low birth weight in the developed countries was 8.1% in 2020, the prevalence in South Asia was 24.9% according to the statistics of 2020 [8]. Moreover, around 90% of the extremely preterm babies are born in low-income countries [9]. The risk of these adverse birth outcomes increases significantly with the presence of chronic hypertension [10]. Evidence suggests that the rate of premature delivery and low birth weight is higher among mothers suffering from chronic hypertension [11].

The incidence and prevalence of chronic hypertension in pregnancy have country-wise variation, but approximately 3–5% of pregnancies worldwide are estimated to be complicated by chronic hypertension [12]. The prevalence of hypertension in Bangladesh is on the rise, being on the verge of epidemiological transition. Around 23% of women of reproductive age suffer from hypertension, and around 40% of them are unaware of their hypertensive status [13]. This poses a high risk of their pregnancy being complicated by chronic hypertension. In Bangladesh, around 41.5% of women experience high-risk pregnancies, including multiple health-related complications [14]. Hypertension during pregnancy manifests as one of the most common forms of health-related issues and elicits a wide array of risks to fetal and maternal health [15,16]. Different studies have identified different sociodemographic factors, maternal age, presence of complications during pregnancy, neonatal factors, and socioeconomic and health system-related factors [17–19] to be associated with adverse birth outcomes in mothers with chronic hypertension [6]. Despite the rising prevalence of hypertension and the proven adverse effect of chronic hypertension on birth outcomes, it is also worth noting that, to our knowledge, no studies have been conducted in Bangladesh focusing on the effect of chronic hypertension on birth outcomes. A few hospital-based studies and some population-based studies in Bangladesh were conducted focusing on pregnancy-induced hypertension [20,21]. Thus, there exists a research gap worth studying. Therefore, this study aims to find the prevalence and factors associated with adverse birth outcomes among women with chronic hypertension who delivered in health facilities.

This research is a step in this very direction of fulfilling the knowledge gap regarding the effect of chronic hypertension in pregnancy on birth outcomes. The findings from this study will also help to emphasize the urgency of care of the chronic hypertensive pregnant women and help the caregivers and policymakers to appreciate the increased risk of the condition, not only the perinatal risk, but also the future risk to both mother and the baby.

## Methodology

### Study area and population

The study was conducted in the center of Rangpur, Bangladesh. Rangpur is one of the major northern cities and the second-largest city corporation in Bangladesh. Rangpur has a population of 708,570 and one of Bangladesh’s highest literacy rates (72.08% among those aged 7 years and above). The study site was in a hospital para in Rangpur and was chosen based on the availability of the maternal and childcare hospital and easy access for both urban and rural residents of Rangpur.

The population for this study was women delivered in the selected hospitals within the study area and were approached irrespective of their place of residence, religion, or socioeconomic status.

### Study design and setting

A multi-center hospital-based cross-sectional study was conducted in 3 different maternity and child hospitals in Rangpur city. The hospitals were chosen purposively based on the average number of patients per month and cooperation from the gynecologists. Participants with diagnosed chronic hypertension and who had c-sections in the selected hospitals within the last 6 months were recruited for the study between June 2023 and August 2023.

### Eligibility criteria

#### Inclusion criteria.

1. Participants aged 18 years and above
2. Participants diagnosed with chronic hypertension
3. Participants delivered their last baby in the selected hospital
4. Participants delivered their last baby via C-section in the last 6 months

#### Exclusion criteria.

1. Unwilling to participate in the study
2. Participants visiting for health conditions unrelated to their last delivery

### Sample size determination

We estimated the sample size for the study using Cochrane’s formula (formula-1) with the following assumptions


$$\text{n}=\frac{z²p(\text{1}-p)}{d²}$$ (1)

where n is the expected sample size, z is the test statistic value corresponding to the 95% level of confidence, which is 1.96, p is the prevalence, which was considered 21.5% from a previous study [22], and d is the margin of error, which was considered 5% for this study. The estimated sample size with a 10% drop-out rate was 290 (rounded), however, we approached more individuals than required. The final sample size for the study was 342.

### Sampling technique and procedure

Gynecologists in the selected hospital were approached to facilitate participant recruitment for the study. Interested gynecologists were instructed to inform the research team if any patient with chronic hypertension who underwent a C-section in the last 6 months visited their hospital chamber for their regular visit during the study period. All women satisfying the criteria were approached for the study. Women who signed the informed written consent form were included in the study. We approached a total of 428 patients from the 3 selected hospitals, and 342 participants provided complete information, making the response rate 80%. Participants were selected conveniently from the selected hospitals and we ensured equal distribution (114 participants from each hospital). Non-random sampling technique was used due to the absence of the sampling frame (institutional database) in the selected hospital.

### Data collection procedure

A pretested semi-structured questionnaire was used, where the questions were selected and organized based on a thorough literature review. The questionnaire was administered by trained data collectors, and pretesting was done among 10 participants conveniently selected from the pre-selected hospitals who were later excluded from the final sample. Information on the sociodemographic characteristics of the participants, family history, chronic disease status, gynecological and obstetric history, and adverse birth outcomes were observed.

### Outcome variable

The outcome variable for the study was the adverse birth outcome among females with chronic hypertension. The measure of the adverse birth outcome was self-reported. The participants were asked if they had experienced any adverse birth outcomes (Prematurity, Small for Gestational Age (SGA), Large for Gestational Age (LGA), Stillbirths, Shoulder dystocia, Intra-Uterine Growth Retardation (IUGR), and Intra-Uterine Fetal Death (IUFD)) during their last pregnancy. All the responses were binary (yes, no) and the participants who responded yes to any of the options were considered to have an adverse birth outcome. Participants with adverse birth outcomes were coded as 1 and no adverse birth outcomes were coded as 0.

### Independent variables

Independent variables considered in this study were: age (≤ 22 years, 23–25 years, 26–28 years, and 29 years and above), educational status of the participants (up to secondary and higher), working status of the participants (working and not working), family monthly income (20,000 or less BDT/month, > 20000–30000 BDT/ month, and >30,000 BDT/month), total number of children (1–3 babies and >3 babies). For the chronic disease status, the participants were asked if they had any chronic diseases other than hypertension. The participants responding yes to any of the chronic diseases from the given list (Heart disease, Asthma, COPD, Chronic kidney disease, Diabetes, Cancer, Stroke, Thyroid disease, PCOS, and musculoskeletal diseases) were coded as 1 and considered to have chronic disease; otherwise coded as 0 and marked to have no other chronic diseases. From the list of complications (Abruption placenta, Placenta Previa, Pre-eclampsia, Eclampsia, GDM, Growth retardation, Low amniotic fluid, PROM, UTI, Birth canal Infection, and PPH) and danger signs (Excessive fever or weakness, Excessive headache or blurred vision, Respiratory distress, Severe abdominal pain, Less movement of the baby, Vaginal bleeding, and Eclampsia or senselessness) for the current pregnancy the participants were asked to respond to all options that applied to them during the interview. The responses were recorded as binary. Participants responding positively to any of the complications or danger signs were considered to have complications or danger signs in the current pregnancy, respectively. Response for the variable, exposure to second-hand smoking during the last pregnancy (were you exposed to second-hand smoking during your last pregnancy?) was binary (yes, no) and was coded 1 for the former and 0 for the latter.

### Data quality control

The overall quality control of the data was supervised and monitored by the principal investigator. The field supervisor was in charge of ensuring the quality of data collection at the field level and reported the daily update back directly to the principal investigator. During the data entry, the research assistants were in charge of overseeing the process. After entry, the data was checked by the research assistant and rechecked by the statistician for data inconsistency. Data cleaning and analysis was supervised by the principal investigator.

### Operational definitions

**Chronic Hypertension:** The American Heart Association (AHA) and American College of Cardiology (ACC) diagnose chronic hypertension when systolic blood pressure is > 130 mm of Hg and/or diastolic blood pressure is > 80 mm of Hg [23].

**Chronic Hypertension in Pregnancy**: Systolic blood pressure >130 mm of Hg and diastolic blood pressure >90 mm of Hg before being pregnant or detected before 20 weeks of pregnancy is defined as chronic hypertension in pregnancy by American College of Obstetrics and Gynecology (ACOG) [24]. This definition was used to define the study participants.

**Anti-HTN medication history:** Medication prescribed by the physician or taken by the patients for hypertension control is defined as the anti-hypertension medication history.

### Data processing and analysis

For the current study, both descriptive and inferential statistics were generated. The frequency distribution of the independent variables was shown as part of the descriptive analysis. Pearson’s chi-square test was calculated to show the association between the independent and outcome variables. Before conducting regression analysis, a test for multicollinearity was conducted to confirm whether there were any highly correlated independent variables. A VIF value of 10 was used as a cut point to determine the multicollinearity among the independent variables. No significant collinearity was observed. Both the bivariate and multivariable logistic regression analyses were applied and all the uncorrelated variables were included in the multivariable model. The crude and adjusted odds ratios, along with their corresponding 95% confidence intervals, were reported. For the statistical analysis STATA version 17.0 was used.

### Ethical approval

Ethical clearance for the study was obtained from the Institutional Review Board (IRB) of the North South University (ethical approval reference number: 2023/OR-NSU/IRB/0407) on 30^th^ April 2023. Permission for data collection was obtained from the hospital authorities of all three hospitals. The study’s objectives were demonstrated to each respondent prior to data collection, and informed written consent was obtained from them. We also ensured the anonymity and confidentiality of the participants. The moral principles set down in the 1964 Declaration of Helsinki, and its later changes were followed.

## Results

### Sociodemographic and lifestyle characteristics of the study population

In our study, the participants’ ages ranged from 18 years to 39 years, with a mean of 26.4 and a standard deviation of 4.4. The highest number of participants belonged to the 26–28 years’ age group (31.3%). The majority of the study participants (72.8%) were found to have completed higher secondary education. Among the study participants, 71.1% were urban residents and only 28.9% were rural residents. Most of the participants (89.2%) belonged to the non-working group. More than 70% of the study participants reported their monthly income to be 30000 BDT or less per month. Only 17.3% of the participants were found to be suffering from chronic illnesses other than hypertension. Complications in current pregnancy were reported by almost half (49.4%) of the participants. Around 18.1% of the study participants reported having danger signs present in the current pregnancy. Most of the participants (94.1%) had no exposure to second-hand smoke during their last pregnancy.

### Prevalence of adverse birth outcome

The overall prevalence of adverse birth outcomes among the study participants was 36% (95% CI: 30.87, 41.30). The prevalence of adverse birth outcomes varied among the participants of different age groups, with a higher prevalence among the age group of 26–28 years (41.1%), followed by 38.8% in the age group 29 years and above (Table 1). Data in Table 1 suggest that the level of educational attainment by the study participants, their place of residence, and working status do not significantly influence the likelihood of adverse birth outcomes. A significantly higher prevalence (52.8%) of adverse birth outcomes was observed among the participants with a family monthly income of more than 30000 BDT. Although no statistical significance was observed, the prevalence of adverse birth outcomes was found to be higher among the participants with 3 or more live births (54.5%) compared to those having 1–3 live births (35.3%). Similarly, participants with chronic diseases (other than hypertension) were found to have a higher prevalence of adverse birth outcomes (45.8%) compared to their counterparts with no chronic disease other than hypertension as shown in Table 1. Respondents who had complications in their current pregnancy (45.6%) or had at least one danger sign of pregnancy present during their current pregnancy (46.8%) were found to have a higher rate of adverse birth outcomes compared to their counterparts (Table 1). It was evident from Table 1 that the prevalence of adverse birth outcomes was nearly double (65%) among the participants with exposure to second-hand smoke during pregnancy compared to their non-exposed counterparts (34.2%).

**Table 1:** Distribution of sociodemographic characteristics and their association to adverse birth outcomes among the study participants.

Variable	Total	Adverse birth outcome		P value
		No (64%)	Yes (36%)	
**Age of the respondents**				
<=22 years	75 (21.9)	53 (70.7)	22 (29.3)	0.288
23-25 years	62 (18.1)	43 (69.4)	19 (30.6)	
26-28 years	107 (31.3)	63 (58.9)	44 (41.1)	
29 years and above	98 (28.7)	60 (61.2)	38 (38.8)	
**Education of the respondent**				
Up to Secondary	93 (27.2)	67 (72)	26 (28)	0.059
Higher secondary and above	249 (72.8)	152 (61)	97 (39)	
**Place of residence**				
1. Urban	243 (71.1)	151 (62.1)	92 (37.9)	0.253
2. Rural	99 (28.9)	68 (68.7)	31 (31.3)	
**Working Status**				
Not working	305 (89.2)	195 (63.9)	110 (36.1)	0.911
Working	37 (10.8)	24 (64.9)	13 (35.1)	
**Family income**				
20000 or less	130 (38)	95 (73.1)	35 (26.9)	**<0.001**
>20000-30000	123 (36)	82 (66.7)	41 (33.3)	
>30000	89 (26)	42 (47.2)	47 (52.8)	
**Total number of children**				
1-3 babies	331 (96.8)	214 (64.7)	117 (35.3)	0.192
>3 babies	11 (3.2)	5 (45.5)	6 (54.5)	
**Chronic disease**				
1. Yes	59 (17.3)	32 (54.2)	27 (45.8)	0.085
2. No	283 (82.7)	187 (66.1)	96 (33.9)	
**Complication in the current pregnancy**				
No	173 (50.6)	127 (73.4)	46 (26.6)	**<0.001**
Yes	169 (49.4)	92 (54.4)	77 (45.6)	
**Danger signs in current pregnancy**				
Yes	62 (18.1)	33 (53.2)	29 (46.8)	**0.05**
No	280 (81.9)	186 (66.4)	94 (33.6)	
**Exposure to secondhand smoking**				
Yes	20 (5.9)	7 (35)	13 (65)	**0.005**
No	319 (94.1)	210 (65.8)	109 (34.2)	

### Distribution of adverse birth outcomes

**Fig 1:**
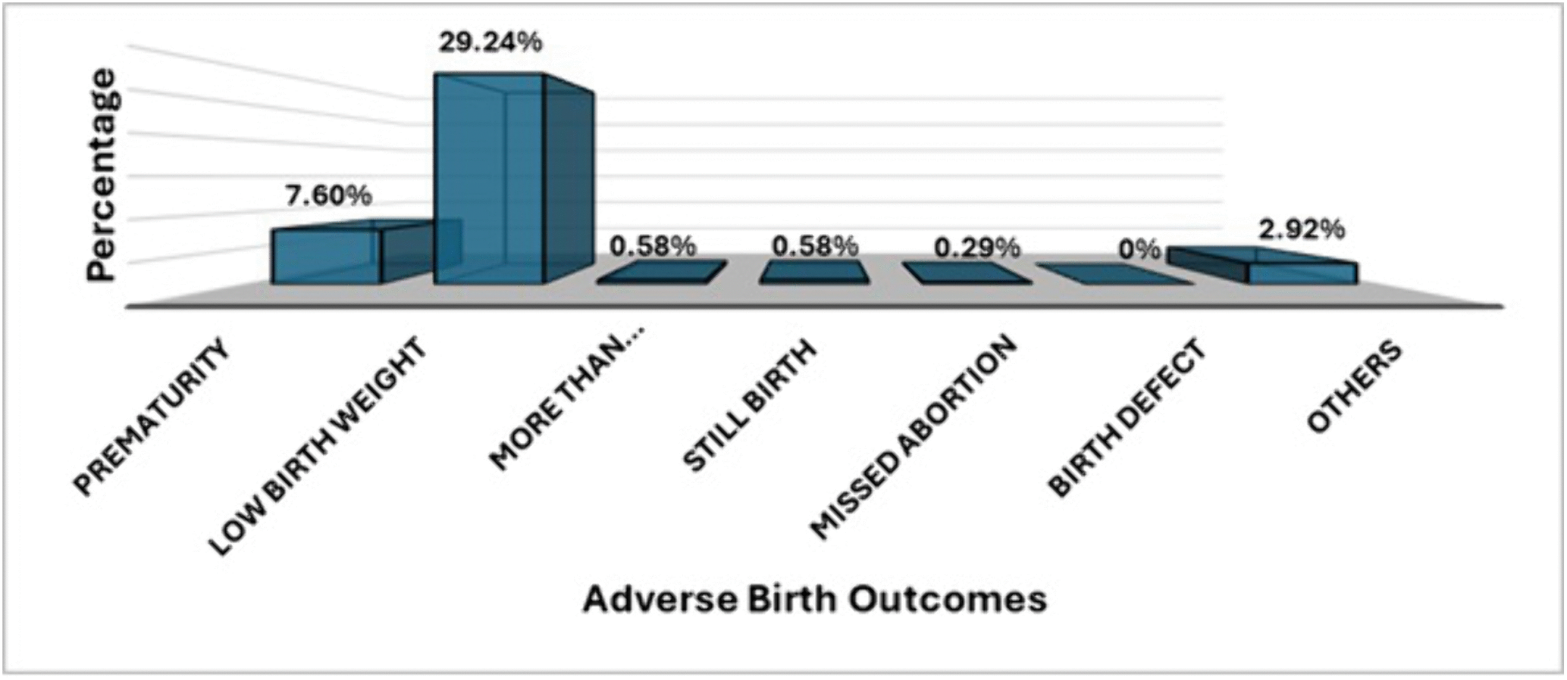
Distribution of adverse birth outcomes.

Fig 1 shows the distribution of adverse birth outcomes among women with chronic hypertension. Among the different adverse outcomes, low birth weight (29.24%) was the most common adverse outcome, followed by prematurity (7.60%). No participants reported birth defects as adverse birth outcomes. More than normal-weight babies, stillbirths, and missed abortions were reported individually by less than 1% of the participants.

### Factors associated with adverse birth outcomes

On bivariable analysis, family monthly income, complications in the current pregnancy, and exposure to secondhand smoking were found to be significantly associated with adverse birth outcomes at 5% level of significance. Participants belonging to higher-income families, with complications in the recent pregnancy and having exposure to second-hand smoking, were found to have 3.04 (95% CI: 1.72, 5.36), 2.31 (95% CI: 1.47, 3.64), and 3.58 (95% CI: 1.39, 9.23) times higher odds of having adverse birth outcomes, respectively, compared to their counterparts. All the independent variables in the bivariable analysis were included in the multivariable analysis, irrespective of their significance level, as no multicollinearity was present.

Table 2 shows the result of the multivariable logistic regression analysis. Among the study participants, mothers belonging to a family with higher income (> 30,000 BDT) were found to have 3.09 (95% CI: 1.51, 6.30) times higher odds of having an adverse birth outcome compared to mothers belonging to a family with a monthly income 20,000 BDT or less. The mothers with chronic diseases along with chronic hypertension and mothers with more than 3 live-born babies were 1.20 (95% CI: 0.64, 2.25) and 3.53 (95% CI: 0.93, 13.40) times higher odds to have adverse birth outcomes compared to their respective counterparts, evident from Table 2. Around 2.55 times higher odds (95% CI: 1.55, 4.21) of having adverse birth outcomes were found among the participants with complicated pregnancies compared to their counterparts with uneventful pregnancies other than chronic hypertension (Table 2). Higher odds (OR: 2.21; 95% CI: 1.17, 4.15) of having adverse birth outcomes were also observed among mothers who had at least one danger sign present in their last pregnancy compared to mothers with no danger signs present. It is evident from Table 2 that participants with exposure to secondhand smoke during their pregnancy were 3.33 (95% CI: 1.16, 9.60) times more likely to have adverse birth outcomes compared to mothers without the exposure.

**Table 2:** Logistic regression analysis to assess the factors associated with adverse birth outcomes.

Variable	COR	95% CI	AOR	95% CI
**Age of the respondent**				
<=22 years	1		1	
23-25 years	1.06	0.51, 2.22	1.04	0.45, 2.39
26-28 years	1.68	0.89, 3.16	1.54	0.75, 3.18
29 years and above	1.53	0.80, 2.89	1.33	0.63, 2.79
**Education of the respondent**				
Upto secondary	1		1	
Higher secondary and above	1.64	0.98, 2.76	1.50	0.79, 2.83
**Place of residence**				
Rural	0.75	0.45, 1.23	0.66	0.38, 1.16
Urban	1		1	
**Working status**				
Working	0.96	0.47, 1.96	0.52	0.22, 1.20
Not Working	1		1	
**Family income**				
20000 or less	1		1	
>20000-30000	1.36	0.79, 2.33	1.12	0.60, 2.09
>30000	3.04	**1.72, 5.36**	3.09	**1.51, 6.30**
**Total number of children**				
1-3 babies	1		1	
> 3 babies	2.19	0.66, 7.35	3.53	0.93, 13.40
**Chronic disease**				
No	1		1	
Yes	1.64	0.93, 2.90	1.20	0.64, 2.25
**Complications in current pregnancy**				
No	1		1	
Yes	2.31	**1.47, 3.64**	2.55	**1.55, 4.21**
**Danger signs in current pregnancy**				
No	1		1	
Yes	1.74	0.99, 3.04	2.21	**1.17, 4.15**
**Exposure to secondhand smoking**				
No	1		1	
Yes	3.58	**1.39, 9.23**	3.33	**1.16, 9.60**

Key: COR: Crude Odds Ratio, AOR: Adjusted Odds Ratio, 95% CI: 95% Confidence Interval.

## Discussion

The prevalence of adverse birth outcomes among women with chronic hypertension who had an institutional delivery was found to be 36% (95% CI: 30.87, 41.30). This finding was consistent with the findings of the studies conducted previously in Ethiopia [25,26], higher than in the study conducted in Tanzania [27]. The possible reason behind this discrepancy in the findings could be the variation in geographical location and demographics of the study participants. Besides the quality of maternal care, the availability of the facility, and other logistic parameters might also contribute to the difference in the findings [25].

In contrast to the previously conducted studies in Bangladesh [28–31], our study observed a higher prevalence of adverse birth outcomes among hypertensive women. While other studies defined ABOs, focusing separately on low birth outcomes, prematurity, or stillbirth separately as an adverse birth outcome [28–31], this study adopted a broader definition encompassing the presence of any of these outcomes as adverse birth outcomes. The difference in the definition of adverse birth outcomes in different studies could be a possible explanation for the differences. On the other hand, all of the studies conducted previously in Bangladesh were among women with no previous history of hypertension [28,29,31]. This study focused on pregnant women with chronic hypertension, which could be another reason behind the higher prevalence of adverse birth outcomes. Institution-based study in a tertiary center could be another plausible explanation behind the higher prevalence of adverse birth outcomes among mothers with chronic hypertension, as women with potential adversity usually show up at the health facility.

Adverse birth outcomes among hypertensive pregnant women in Rangpur were found to vary by family income. Women belonging to high-income families were found to have higher odds of having at least one adverse birth outcome compared to their counterparts, suggesting that socioeconomic factors are one of the influential factors in developing adverse birth outcomes among hypertensive mothers. The probable explanation behind this finding could be the choice of a sedentary lifestyle and a high-calorie diet among the high-income family members, which contributes to their high BMI [32]. Mothers with high BMI were found to have higher odds of having pregnancy complications [33,34], which in turn might give rise to potential adverse birth outcomes [35]. This finding further reinforces the previous claims among hypertensive women.

The risk of adverse birth outcomes was found to be higher among hypertensive mothers with complicated pregnancies compared to mothers with uneventful pregnancies other than chronic hypertension. This finding is in line with previous studies [25,36–39]. Pregnancy complications like premature rupture of the membrane, eclampsia, and infections are well-established factors that contribute to the development of adverse birth outcomes [40–43]. A previous study conducted in Ethiopia suggests that complications during pregnancy might affect the fetal well-being in the uterus [25]. The possible reason behind this finding could be that pregnancy complications cause fetal distress, which might lead to adverse birth outcomes [37]. This finding further highlights how chronic hypertension affects the birth outcomes of a complicated pregnancy and strengthens the previous findings. Therefore, early detection and treatment of high-risk and complicated pregnancies could be safe and cost-effective strategies to reduce adverse birth outcomes, especially among mothers with chronic hypertension.

Higher odds of adverse birth outcomes were found among the mothers with danger signs present during their last pregnancy. A similar finding was also found in previous studies conducted in Kenya [6], Denmark [44], and among countries in Sub-Saharan Africa [3]. Evidence suggests that danger signs of pregnancy pose a serious risk to the mother and the developing fetus [45,46] including disruption in the development of the nervous system and cognitive functions [47,48]. This evidence might be considered a contributing factor to our study findings. Vigilant and timely interventions in pregnancies with danger signs among women with chronic hypertension can reduce the prevalence of adverse birth outcomes among those mothers.

Hypertensive pregnant women with exposure to secondhand smoke during their pregnancy were found to be at higher risk of experiencing adverse birth outcomes compared to non-exposed women. This finding was in line with the studies conducted in India [49], Malaysia [50], and Saudi Arabia [51]. The possible explanation behind this finding could be the damage to the placenta due to the rising maternal cytokine level or reduced placental blood flow due to inhaled carbon monoxide and nicotine [52]. These biological reasons could be the possible reasons behind the increased risk of adverse birth outcomes among mothers exposed to secondhand smoke.

## Strengths and limitations

The current study portrays a scenario of the effect of chronic hypertension on pregnancy outcomes, which is the first and only study conducted in Bangladesh. The finding of this study fills an important knowledge gap about the effect of chronic hypertension on pregnancy outcomes in Bangladeshi women. The rigorous analytical technique provides a robust result in identifying the factors associated with adverse birth outcomes among women with chronic hypertension.

The study has a few limitations. Firstly, due to the cross-sectional nature of the study, we could not establish any causal association with adverse birth outcomes among the participants. Secondly, the study being conducted in the health facility could affect the prevalence rate. Thirdly, being an institutional-based study conducted in a tertiary center and having patient with other co-morbidities and danger signs along with the chronic hypertension limits the generalizability of the study findings. Fourthly, the study might be prone to the effect of some unknown confounding variables. Lastly, the self-reported nature of the response to the questions could lead to recall bias.

### Policy implication

Continuous screening for factors associated with high-risk pregnancies during the inter-conception period could be an effective approach towards early identification of the risk factors and prevention of the development of adverse birth outcomes. Steps should be taken to educate not only pregnant mothers but also reproductive-aged couples to create awareness about the effect of chronic hypertension on pregnancy and birth outcomes. Measures should also be taken to mitigate exposure to secondhand smoke. Appropriate measures to reduce the household smoke exposure risk from cooking fuel could be an effective strategy in this regard.

## Conclusion and recommendation

This study observed a high prevalence of adverse birth outcomes among pregnant women with chronic hypertension who had an institutional delivery. Family income, complications during pregnancy, secondhand smoke exposure, and the presence of danger signs during pregnancy were found to be significantly associated with adverse birth outcomes. The finding is concerning and requires immediate community-based action focused on the important variables to reduce both maternal and child mortality. To reduce the incidence of adverse birth outcomes, mothers with complications during the pregnancy period should be given special attention throughout the pregnancy period. A multifaceted approach should be adopted and tailored for pregnant women with chronic hypertension to reduce the prevalence of adverse birth outcomes. Policies should be developed for a smoke-free home, especially during the pregnancy period, to avoid the development of adverse birth outcomes. Further longitudinal studies are warranted to identify any causal association between vital variables and adverse birth outcomes.

## Supporting information

S1 Table Adverse birth outcome. (DOCX)

S1 File Data and questionnaire files. (ZIP)

S2 File STROBE checklist file. (DOCX)

## Abbreviations

ABO: Adverse Birth Outcome
LMIC: Low- and Middle-Income Countries
LBW: Low Birth Weight
BDT: Bangladeshi Taka
AOR: Adjusted Odds Ratio.
