# Supplementary material for: Prevalence and factors associated with adverse birth outcomes among women with chronic hypertension in Rangpur: A multi-center cross-sectional study
Source: PLoS One. 2025 Dec 11;20(12):e0337526. doi: 10.1371/journal.pone.0337526 (PMC12697937; doi:10.1371/journal.pone.0337526)
Supplement: S1 Table — (DOCX) [file pone.0337526.s001.docx]

**Supplementary Table 1: Adverse birth outcome**

| **Adverse birth outcome** | **Frequency** | **Percentage** |
| --- | --- | --- |
| No adverse birth outcome | 219 | 64.04% |
| 1 Adverse birth outcome | 108 | 31.58% |
| 2 Adverse birth outcomes | 12 | 3.51% |
| >2 Adverse birth outcomes | 3 | 0.88% |

The table shows the frequency and percentage of adverse birth outcome during their recent delivery reported by the participating women. Among the study participants, 64.04% of the women had no adverse birth outcome during their recent birth. Only 1 adverse effect was reported by 31.58% of the women during the survey. About 4.39% of the participants were found to have 2 or more adverse birth outcome during their last delivery.
