# Supplementary material for: Prevalence and factors associated with adverse birth outcomes among women with chronic hypertension in Rangpur: A multi-center cross-sectional study
Source: PLoS One. 2025 Dec 11;20(12):e0337526. doi: 10.1371/journal.pone.0337526 (PMC12697937; doi:10.1371/journal.pone.0337526)
Supplement: S1 File — (ZIP) [file pone.0337526.s002.zip › Data_Questionnaire/ABO_SurveyQuestionnaire.docx]

**Questionnaire (English)**

| **ID:** | | | |
| --- | --- | --- | --- |
| **Module 1: Sociodemographic Information of the Respondent** | | | |
| **Question** | | **Option** | **Answer** |
| 1.1 | Age (in years) |  |  |
| 1.2 | The highest level of education attained | 1. No education 2. Primary 3. Secondary 4. Higher |  |
| 1.3 | Place of residence | 1. Urban 2. Rural |  |
| 1.4 | Blood pressure (in mm of Hg) |  |  |
| 1.5 | Working status | 1. Working 2. Not working |  |
| 1.6 | Monthly income |  |  |

| **Module 2: Co-morbidities** | | | |
| --- | --- | --- | --- |
| **Question** | | **Option** | **Answer** |
| 2.1 | Have you ever been diagnosed with any chronic illnesses?  If “yes” continue to the next question  If “no” skip to the next module | 1. Yes 2. No |  |
| 2.2 | Which of the illnesses have you been diagnosed with?  Choose all that apply | 1. Diabetes 2. Heart disease 3. Chronic respiratory disease (asthma, COPD) 4. Chronic kidney disease 5. Cancer 6. Stroke 7. Thyroid disease 8. PCOS 9. Musculoskeletal disease 10. Others |  |

| **Module 3: Gynecological and Obstetric Information** | | | |
| --- | --- | --- | --- |
| **Question** | | **Option** | **Answer** |
| 3.1 | Total number of children ever born |  |  |
| 3.2 | What was the gestational age of the baby at the time of delivery? (in weeks) |  |  |
| 3.3 | What was the weight of the baby at delivery (in gm) |  |  |
| 3.4 | Were you exposed to secondhand smoke during your recent pregnancy? | 1. Yes 2. No |  |
| 3.5 | Have you experienced pregnancy complications in your recent pregnancy?  If “yes” continue to the next question  If “no” skip to question 3.7 | 1. Yes 2. No |  |
| 3.6 | Which one of these complications have you experienced?  Choose all that apply  Code:  If “yes” then 1  If “no” then 0 | Abruption placenta |  |
|  |  | Placenta previa |  |
|  |  | Pre-eclampsia |  |
|  |  | Eclampsia |  |
|  |  | Gestational Diabetes |  |
|  |  | Growth retardation |  |
|  |  | Low amniotic fluid |  |
|  |  | PROM |  |
|  |  | Premature PROM |  |
|  |  | UTI |  |
|  |  | Birth canal infection |  |
|  |  | PPH |  |
|  |  | Others |  |
| 3.7 | Have you experienced any of these danger signs during your last pregnancy?  Choose all that apply  Code:  If “yes” then 1  If “no” then 0 | High fever or weakness |  |
|  |  | Severe headache or blurred vision |  |
|  |  | Respiratory distress |  |
|  |  | Abdominal pain |  |
|  |  | Vaginal bleeding |  |
|  |  | Less movement of the baby |  |
|  |  | Eclampsia or senselessness |  |
| 3.8 | Have you ever experienced adverse birth outcomes in your recent pregnancy?  If “yes” continue to the next question  If “no” end the survey | 1. Yes 2. No |  |
| 3.9 | Which of these adverse birth outcomes have you experienced?  Choose all that apply  Code:  If “yes” then 1  If “no” then 0 | Prematurity |  |
|  |  | SGA |  |
|  |  | LGA |  |
|  |  | Stillbirth |  |
|  |  | Missed abortion |  |
|  |  | Birth defect |  |
|  |  | Others (IUGR, IUFD) |  |
