# Supplementary material for: Prevalence and factors associated with adverse birth outcomes among women with chronic hypertension in Rangpur: A multi-center cross-sectional study
Source: PLoS One. 2025 Dec 11;20(12):e0337526. doi: 10.1371/journal.pone.0337526 (PMC12697937; doi:10.1371/journal.pone.0337526)
Supplement: S2 File — (DOCX) [file pone.0337526.s003.docx]

STROBE Statement—checklist of items that should be included in reports of observational studies

|  | Item No | Recommendation |
| --- | --- | --- |
| **Title and abstract** | 1 | (*a*) Indicate the study’s design with a commonly used term in the title or the abstract **Abstract, subsection: Methods, Page 1** |
|  |  | (*b*) Provide in the abstract an informative and balanced summary of what was done and what was found **Abstract, subsection: Methods and Results, Page 1** |
| Introduction | | |
| Background/rationale | 2 | Explain the scientific background and rationale for the investigation being reported  **Introduction, Page 2** |
| Objectives | 3 | State specific objectives, including any prespecified hypotheses  **Introduction, Page 3** |
| Methods | | |
| Study design | 4 | Present key elements of study design early in the paper  **Methods, subsection: Study Area and Population, Page 3** |
| Setting | 5 | Describe the setting, locations, and relevant dates, including periods of recruitment, exposure, follow-up, and data collection  **Methods, subsection: Study Area and Population, Page 3** |
| Participants | 6 | (*a*) *Cohort study*—Give the eligibility criteria, and the sources and methods of selection of participants. Describe methods of follow-up  *Case-control study*—Give the eligibility criteria, and the sources and methods of case ascertainment and control selection. Give the rationale for the choice of cases and controls  *Cross-sectional study*—Give the eligibility criteria, and the sources and methods of selection of participants  **Methods, subsection: Study Design and Setting and Eligibility Criteria, Page 4** |
|  |  | (*b*) *Cohort study*—For matched studies, give matching criteria and number of exposed and unexposed **Not applicable**  *Case-control study*—For matched studies, give matching criteria and the number of controls per case **Not applicable** |
| Variables | 7 | Clearly define all outcomes, exposures, predictors, potential confounders, and effect modifiers. Give diagnostic criteria, if applicable  **Methods, subsection: Outcome Variables, Page 5-6**  **Methods, subsection: Independent Variables, Page 6** |
| Data sources/ measurement | 8* | For each variable of interest, give sources of data and details of methods of assessment (measurement). Describe comparability of assessment methods if there is more than one group  **Methods, subsection: Outcome Variables, Page 5-6**  **Methods, subsection: Independent Variables, Page 6** |
| Bias | 9 | Describe any efforts to address potential sources of bias  **Not applicable** |
| Study size | 10 | Explain how the study size was arrived at  **Methods, subsection: Sample Size Determination, Page 4** |
| Quantitative variables | 11 | Explain how quantitative variables were handled in the analyses. If applicable, describe which groupings were chosen and why  **Methods, subsection: Independent Variables, Page 6** |
| Statistical methods | 12 | (*a*) Describe all statistical methods, including those used to control for confounding  **Methods, subsection: Data Processing and Analysis, Page 7** |
|  |  | (*b*) Describe any methods used to examine subgroups and interactions **Not applicable** |
|  |  | (*c*) Explain how missing data were addressed **Not applicable** |
|  |  | (*d*) *Cohort study*—If applicable, explain how loss to follow-up was addressed  *Case-control study*—If applicable, explain how matching of cases and controls was addressed  *Cross-sectional study*—If applicable, describe analytical methods taking account of sampling strategy  **Methods, subsection: Data Processing and Analysis, Page 7** |
|  |  | (*e*) Describe any sensitivity analyses  **Methods, subsection: Data Processing and Analysis, Page 7** |

| Results | | |
| --- | --- | --- |
| Participants | 13* | (a) Report numbers of individuals at each stage of study—eg numbers potentially eligible, examined for eligibility, confirmed eligible, included in the study, completing follow-up, and analysed **Methods, subsection: Sampling Technique and Procedure, Page 5** |
|  |  | (b) Give reasons for non-participation at each stage  **Methods, subsection: Sampling Technique and Procedure, Page 5** |
|  |  | (c) Consider use of a flow diagram  **Methods, subsection: Sampling Technique and Procedure, Page 5** |
| Descriptive data | 14* | (a) Give characteristics of study participants (eg demographic, clinical, social) and information on exposures and potential confounders **Results, subsection: Sociodemographic and Lifestyle Characteristics of the study Participants, Page 8** |
|  |  | (b) Indicate number of participants with missing data for each variable of interest **Not applicable** |
|  |  | (c) *Cohort study*—Summarise follow-up time (eg, average and total amount) |
| Outcome data | 15* | *Cohort study*—Report numbers of outcome events or summary measures over time  **Not applicable** |
|  |  | *Case-control study—*Report numbers in each exposure category, or summary measures of exposure **Not applicable** |
|  |  | *Cross-sectional study—*Report numbers of outcome events or summary measures **Results, subsection: Sociodemographic and Lifestyle Characteristics of the study Participants, Page 8** |
| Main results | 16 | (*a*) Give unadjusted estimates and, if applicable, confounder-adjusted estimates and their precision (eg, 95% confidence interval). Make clear which confounders were adjusted for and why they were included **Results, subsection: Prevalence of Adverse Birth Outcome, Page 9 Results, subsection: Factors Associated with Adverse Birth Outcomes, Page 10-11** |
|  |  | (*b*) Report category boundaries when continuous variables were categorized **Results, subsection: Prevalence of Adverse Birth Outcome, Page 9**  **Methods, subsection: Independent Variables, Page 6** |
|  |  | (*c*) If relevant, consider translating estimates of relative risk into absolute risk for a meaningful time period **Not applicable** |
| Other analyses | 17 | Report other analyses done—eg analyses of subgroups and interactions, and sensitivity analyses **Not Applicable** |
| Discussion | | |
| Key results | 18 | Summarise key results with reference to study objectives **Discussion, First paragraph, Page 12** |
| Limitations | 19 | Discuss limitations of the study, taking into account sources of potential bias or imprecision. Discuss both direction and magnitude of any potential bias **Strengths and Limitations, Page 14-15** |
| Interpretation | 20 | Give a cautious overall interpretation of results considering objectives, limitations, multiplicity of analyses, results from similar studies, and other relevant evidence **Discussion, Page 12-14** |
| Generalisability | 21 | Discuss the generalisability (external validity) of the study results |
| Other information | | |
| Funding | 22 | Give the source of funding and the role of the funders for the present study and, if applicable, for the original study on which the present article is based |

*Give information separately for cases and controls in case-control studies and, if applicable, for exposed and unexposed groups in cohort and cross-sectional studies.

**Note:** An Explanation and Elaboration article discusses each checklist item and gives methodological background and published examples of transparent reporting. The STROBE checklist is best used in conjunction with this article (freely available on the Web sites of PLoS Medicine at http://www.plosmedicine.org/, Annals of Internal Medicine at http://www.annals.org/, and Epidemiology at http://www.epidem.com/). Information on the STROBE Initiative is available at www.strobe-statement.org.
